# Outcomes of Laparoscopic Versus Open Surgery in Elderly Patients with Rectal Cancer

**DOI:** 10.31557/APJCP.2021.22.4.1325

**Published:** 2021-04

**Authors:** Qi Zhang, Jianwei Liang, Jianan Chen, Shiwen Mei, Zheng Wang

**Affiliations:** *Department of Colorectal Surgery, National Cancer Center/National Clinical Research Center for Cancer/Cancer Hospital, Chinese Academy of Medical Sciences and Peking Union Medical College, China.*

**Keywords:** Laparoscopic surgery, open surgery, rectal cancer, elderly patients

## Abstract

**Background:**

Laparoscopic colorectal resection has been gaining popularity over the past two decades-and the number of elderly patients with colorectal cancer treated with a surgical modality has gradually increased. However, studies about laparoscopic rectal surgery in elderly patients with long-term oncologic outcomes are limited. In this study, we evaluated the safety and effectiveness of laparoscopic resection in patients with rectal cancer aged ≥80 y.

**Methods:**

From 2007-2015, a total of 84 consecutive patients with rectal cancer from a single institution were included, 45 patients undergoing laparoscopic rectal resection were compared with 39 patients undergoing open rectal resection.

**Results:**

The two groups were well balanced in terms of age, gender, body mass index, American society of anesthesiologists scores, previous abdominal surgery, neoadjuvant therapy, tumor stage, distance of tumor from the anal verge, and comorbidities. One (2.2%) patient in the laparoscopic group required conversion to open surgery. Laparoscopic surgery was associated with signiﬁcantly longer operating time (160.1±28.2 versus 148.2±41.3 min; P=0.031), less intraoperative blood loss (80.5±20.9 versus 160.3±42.4 mL; P=0.002), less need of blood transfusion (6.7% versus 20.5%; P=0.003), a shorter time to diet recovery (2.5±1.5 versus 4.9±1.1; P=0.015) and postoperative hospital stay (7.5±4.5 versus 10.8±4.2; P=0.035), lower overall postoperative complication rate (8.9% versus 20.5%; P=0.017), and wound-related complication rate (4.4% versus 10.2%; P=0.013) when compared with open surgery. Specimen length, no. of retrieced lymph nodes, positive distal and circumferential margin rate, mortality rate, and reoperation rate were not signiﬁcantly different between two groups. The disease-free and overall 5-year survival rates were similar between two groups.

**Conclusions:**

Laparoscopic rectal surgery is safe and feasible in patients aged≥80 y and is associated with similar long-term oncologic outcomes when compared with open surgery.

## Introduction

Colorectal cancer is the one of the most common malignancies worldwide, and in China colorectal cancer is the fourth most common cancer (Gu and Chen, 2013a). The incidence rate of colorectal cancer increases with the entry of old age, and the number of elderly patients increased gradually. Most of the elderly patients with colorectal cancer complicated with cardiopulmonary disease (Turrentine et al., 2006), whether to accept laparoscopic surgery is a problem. Laparoscopic colorectal resection has become popular during the past two decades. A series of randomized, prospective clinical trials have conﬁrmed that laparoscopic surgery for colorectal cancer is associated with better immune and inﬂammatory response, short hospitalization, more rapid postoperative recovery, and equally long-term oncologic outcomes compared with open surgery (Fleshman et al., 2007; Lacy et al., 2008; Buunen et al., 2009; Green et al., 2013; van der Pas et al., 2013a; Jeong et al., 2014a; Jeong et al., 2014b; Bonjer et al., 2015). However, the safety and effectiveness of laparoscopic surgery is not clear in octogenarians with rectal cancer who might have comorbidities such as cardiovascular or pulmonary disease. In addition, data on laparoscopic versus open resection in elderly rectal cancer patients with long-term outcomes are limited. In this study, we evaluated the short-term and long-term outcomes of laparoscopic and open resection in rectal cancer patients aged ≥80 y. 

## Materials and Methods

*Patients and methods*

From June 2007 to June 2015, 84 consecutive patients with rectal cancer aged ≥80 who were intended to receive curatively resected surgery in our hospital were studied retrospectively. All the patients were diagnosed before surgery, the patients whose tumors were >15 cm away from the anal verge, with synchronous metastases and those who only had simple stoma formation were excluded. 

All the surgeons in this study perform both laparoscopic and open rectal surgery. All the patients were given the option of laparoscopic and open surgery. The choice of surgical approach was made between patient and surgeon after the risks and benefits of different approaches had been explained adequately. All the patients received computed tomography scan and pelvic magnetic resonance imaging before surgery for preoperative staging. The technique details of laparoscopic surgery for rectal cancer included multiport techniques and TME principle (Kitano et al., 2005). Patients who required conversion were included in the laparoscopic group because data were analyzed according to an intention-to-treat basis. Postoperative managements were the same between laparoscopic and open groups. 

The clinicopathological and operative data were documented, including age, gender, body mass index, American society of anesthesiologists scores, previous abdominal surgery, neoadjuvant therapy, tumor stage, distance of tumor from the anal verge, comorbidities, surgical procedure, histology, specimen length, no. of retrieced lymph nodes, positive distal margin and circumferential margin. Operative procedure details were recorded, including operating time and intraoperative blood loss. Tumors were staged according to the TNM classification of the International Union Against Cancer (UICC) (Sobin and Compton, 2010). Postoperative complications were monitored for 30 days after surgery, mortality was deﬁned as death within 30 days after surgery. Data of last follow-up and vital status were collected on all patients. After hospital discharge, patients were suggested to visit the doctors every three months within ﬁrst two year and every six months for a total of 5 year. The study was conducted with the approval of the ethics committee of our hospital, and we obtained informed consent from all patients before surgery.

*Statistical analysis*

Continuous variables are expressed as median and were analyzed with the ManneWhitney U-test, whereas categorical ones are expressed as percentage value and were analyzed by chi-square test or Fisher exact test when appropriate. Overall survival was deﬁned from the date of operation to the date of death. Recurrence was deﬁned by either imaging studies or pathologic ﬁndings. KaplaneMeier method was used to analyze the survival of patients, and the curve of survival between groups was analyzed by the log-rank test. All statistical tests were two-sided, and a P value of <0.05 was considered statistically signiﬁcant. Data were analyzed by Statistical Package for the Social Science 18.0 for Windows (Statistical Package for the Social Science Inc, Chicago, IL). 

## Results

In our hospital, a total of 84 patients were included in this study, 53.6% (45/84) patients underwent laparoscopic resection and 46.4% (39/84) patients underwent open resection. The clinicopathological characteristics of patients were presented in Table 1. The two groups were well balanced in terms of age, gender, body mass index, American society of anesthesiologists scores, previous abdominal surgery, neoadjuvant therapy, tumor stage, distance of tumor from the anal verge, and comorbidities.

The surgical outcomes of laparoscopic group and open group were detailed in Table 2. Types of operation were not statistically different between groups. One (2.2%) patient in the laparoscopic group required conversion due to adhesion. Laparoscopic surgery was associated with signiﬁcantly longer operating time (160.1±28.2 versus 148.2±41.3 min; P= 0.031), less intraoperative blood loss (80.5±20.9 versus 160.3±42.4 mL; P=0.002), less need of blood transfusion (6.7% versus 20.5%; P=0.003), a shorter time to diet recovery (2.5±1.5 versus 4.9±1.1; P=0.015) and postoperative hospital stay (7.5±4.5 versus 10.8±4.2; P=0.035) when compared with open surgery. Surgical procedure, histology, specimen length, no. of retrieced lymph nodes, positive distal margin and circumferential margin were not statistically different between the two groups. 

The overall postoperative complication rate was signiﬁcantly lower in the laparoscopic group than in the open group (8.9% versus 20.5%; P=0.017), both groups showed similar rate of complication regarding anastomotic leakage, abdominal abscess, intestinal obstruction, urinary retention, urinary tract infection, paralytic ileus, postoperative bleeding, wound dehiscence, and deep vein thrombosis(Table 2). The laparoscopic surgery group showed a significantly lower incidence of wound infection (4.4% versus 10.2%; P=0.013). Mortality rate and reoperation rate was not signiﬁcantly different between the two groups. 

The median follow up was 62 month (range 12-87 month). In the laparoscopic group, median follow-up was 65 month (range 12-87 month) , and 62 month (range 12-76 month) in the open group (P=0.840). Local recurrence was observed in 7 (8.3%) patients, 4 patients in the laparoscopic group, and 3 patients in the open group. Distant recurrent disease was observed in 9 (10.7%) patients, 5 patients in the laparoscopic group, and 4 patients in the open group. The 5-year disease-free survival rate was 67.7% in the laparoscopic group versus 64.1% in the open group (P=0.532). The overall 5-year survival rate was 68.9% in the laparoscopic group and 66.7% in the open group (P=0.617). The difference was not statistically signiﬁcant. 

**Table 1:** Clinicopathological Factors

	Laparoscopic group (n = 45)	Open group (n = 39)	P value
Age (years)	82.6±6.4	81.3±8.7	0.778
Gender (male / female)	25/20	22/17	0.546
BMI(kg/m^2^)	26.5±4.5	25.8±5.2	0.684
ASA score			0.605
1	5 (11.1%)	6 (15.4%)	
2	21 (46.7%)	18 (46.2%)	
3	19 (42.2%)	15 (38.5%)	
Previous abdominal surgery	2 (4.4)	2 (5.1)	0.834
Neoadjuvant	10 (22.2%)	8 (20.5%)	0.684
therapy			
Tumor stage			0.763
I	5 (11.1%)	4 (10.3%)	
II	28 (62.2%)	26 (66.7%)	
III	12 (26.7%)	9 (23.1%)	
Distance of tumor from the anal verge (cm)	7.8±1.7	8.1±3.9	0.596
Comorbidities	36 (80.0%)	29 (74.4%)	0.728

**Table 2:** Surgical Outcomes of Laparoscopic Group and Open Group

	Laparoscopic group (n = 45)	Open group (n = 39)	P
Surgical procedure			0.725
Anterior resection	28 (62.2%)	26 (66.7%)	
Abdominoperineal resection	14 (31.1%)	10 (25.6%)	
Hartmann procedure	3 (6.7%)	3 (7.7%)	
Operation time (min)	160.1±28.2	148.2±41.3	0.031
Intraoperative blood loss (ml)	80.5±20.9	160.3±42.4	0.002
Number of patients transfused	3 (6.7%)	8 (20.5%)	0.003
Differentiation			0.732
Well	8 (17.8%)	6 (15.4%)	
Moderately	12 (26.7%)	14 (35.9%)	
Poor	20 (44.4%)	18 (46.2%)	
Mucinous	5 (11.1%)	1 (2.6%)	
Specimen length (cm)	26.5±4.5	25.5±5.5	0.864
No. of retrieced lymph nodes	18.5±5.5	15.5±7.5	0.832
Positive distal margin	1 (2.2%)	0 (0%)	0.745
Positive circumferential margin	1 (2.2%)	2 (5.1%)	0.852
Time to diet recovery (days)	2.5±1.5	4.9±1.1	0.015
Postoperative hospital stay (days)	7.5±4.5	10.8±4.2	0.035
Postoperative complications	4 (8.9%)	8 (20.5%)	0.017
Infection of abdominal incision	2 (4.4%)	4 (10.2%)	0.013
Anastomotic leak	1 (2.2%)	1 (2.6%)	N.S
Abdominal abscess	0	0	
Intestinal obstruction	1 (2.2%)	1 (2.6%)	N.S
Urinary retention	0	0	N.S
Urinary tract infection	0	1 (2.6%)	N.S
Paralytic ileus	0	0	N.S
Postoperative bleeding	0	1 (2.6%)	N.S
Wound dehiscence	0	0	N.S
Deep vein thrombosis	0	0	N.S
Mortality	1 (2.2%)	1 (2.6%)	N.S
Reoperation	2 (4.4%)	2 (5.1%)	N.S

## Discussion

Colorectal cancer is one of the most common malignant tumors in the world, the incidence rate of colorectal cancer has been increasing in recent 40 years in China (Ouabdelmoumen et al., 2018; Yang et al., 2020). Compared with western countries, rectal cancer is more common than colon cancer in China (Gu and Chen, 2013b). Similar to other malignancies, rectal cancer is also common in older patients. Along with an aging society, the number of elderly patients who received surgical treatment for rectal cancer has gradually increased (Jiang et al., 2009). However, compared with younger patients, most elderly patients have cardiovascular or pulmonary diseases and have reduced functional reserve, which increases the risk of surgery and morbidity (Hentati et al., 2018). 

Several multicenter randomized controlled trials have compared the efficacy of laparoscopic surgery and open surgery in the treatment of colon/rectal cancer. It is confirmed that laparoscopic surgery can reduce postoperative pain, rapidly restore intestinal function, shorten hospital stay compared with open surgery, and have similar long-term prognosis of tumor, such as overall survival rate and disease-free survival rate (Lujan et al., 2009; Kang et al., 2010a; Jeong et al., 2014c; Seshadri et al., 2018). So, recently laparoscopic surgery for colorectal cancer has become common and widely accepted as a therapeutic option, and laparoscopic resection of rectal cancer may also be an effective treatment for elderly patients (Frasson et al., 2008). Previously, some studies have reported the safety and feasibility of laparoscopic surgery in elderly patients with colorectal caner (Robinson et al., 2011; She et al., 2013; Itatani et al., 2018). 

However, the safety and effectiveness of laparoscopic surgery is not clear in octogenarians older than 80 years with rectal cancer who might have comorbidities such as cardiovascular or pulmonary disease. Although some randomized trials have reported the safety and feasibility of laparoscopic surgery in elderly patients (Miyasaka et al., 2014; Inoue et al., 2015), but these reports included septuagenarian or only colon cancer, and laparoscopic surgery for rectal cancer is more challenging than those of colon resection, and studies about laparoscopic rectal surgery in patients older than 80 years with long-term oncologic outcomes are limited. 

In this study, the age, gender, body mass index, American society of anesthesiologists scores, previous abdominal surgery, neoadjuvant therapy, tumor stage, distance of tumor from the anal vergesite, and comorbidities were comparable between laparoscopic surgery and open surgery groups. We observed a significantly faster recovery of bowel function, shorter postoperative hospital stay, less blood loss, fewer overall postoperative and wound-related complications in the group receiving laparoscopic surgery, which is similar to the previous reports comparing laparoscopic with open surgery for colorectal cancer in elderly patients (Guillou et al., 2005; Kang et al., 2010b; van der Pas et al., 2013b). In our study, the percentages of patients who needed blood transfusion was lower in the laparoscopic surgery group than those in the open surgery group. In our study, 45 elderly patients received laparoscopic surgery and conversion to open surgery was required in one cases (2.2%%) of laparoscopic surgery due to extensive adhesion. We found that laparoscopic rectal resection could be safely performed in elderly patients with low rate of conversion and perioperative morbidity.

The oncologic outcomes of laparoscopic surgery on elderly patients with rectal cancer are limited. The anatomic complexity of the pelvis and more technical expertise demands for TME than colectomy. Radicality of resection, as assessed by the number of harvested lymph nodes and the rate of positive resected distal and circumferential margins of the specimen, did not differ between the two groups. So our study demonstrated that laparoscopic surgery was as effective as open approach for rectal cancer, and the principles of TME were well achieved. In this study, the overall 5-year survival rate and the 5-year disease-free survival rate were not significantly different between the laparoscopic group and the open group. Our results demonstrated the safety and feasibility of laparoscopic surgery for elderly patients with rectal cancer and better short-term outcomes and equivalent long-term oncologic outcomes compared with open surgery. 

This study had some limitations. First, this study was not a large-scale multicenter randomized trial, but retrospective study conducted at a single institute. Second, the operative method was different depending on different surgeons who have their own preferences. 

In conclusion, laparoscopic rectal resection is safe and feasible in patients aged ≥80 y and is associated with better short-term outcomes including faster recovery of bowel function, a shorter postoperative hospital stay, less blood loss, reduced overall postoperative and wound-related complications, less need of blood transfusion when compared with open resection. Similar oncological long-term outcomes between the laparoscopic group and the open group clarify the true feasibility of laparoscopic surgery in elderly patients with rectal cancer. Based on these ﬁndings, we suggest that elderly patients with resectable rectal cancer should give priority to laparoscopic surgery. 

## Author Contribution Statement

Zheng Wang and Qi Zhang contributed the conceiving and designing the study. Jianwei Liang and Jianan Chen did the collecting of data. Shiwen Mei did the analyzing and interpreting of data. Qi Zhang and Zheng Wang did the writing of the article. Zheng Wang approved the final version of the article.

## References

[B1] Bonjer HJ, Deijen CL, Abis GA (2015). A randomized trial of laparoscopic versus open surgery for rectal cancer. N Engl J Med.

[B2] Buunen M, Veldkamp R, Hop WC (2009). Survival after laparoscopic surgery versus open surgery for colon cancer: long-term outcome of a randomised clinical trial. Lancet Oncol.

[B3] Fleshman J, Sargent DJ, Green E (2007). Laparoscopic colectomy for cancer is not inferior to open surgery based on 5-year data from the COST Study Group trial. Ann Surg.

[B4] Frasson M, Braga M, Vignali A (2008). Benefits of laparoscopic colorectal resection are more pronounced in elderly patients. Dis Colon Rectum.

[B5] Green BL, Marshall HC, Collinson F (2013). Long-term follow-up of the Medical Research Council CLASICC trial of conventional versus laparoscopically assisted resection in colorectal cancer. Br J Surg.

[B6] Gu J, Chen N (2013a). Current status of rectal cancer treatment in China. Colorectal Dis.

[B7] Gu J, Chen N (2013b). Current status of rectal cancer treatment in China. Colorectal Dis.

[B8] Guillou PJ, Quirke P, Thorpe H (2005). Short-term endpoints of conventional versus laparoscopic-assisted surgery in patients with colorectal cancer (MRC CLASICC trial): multicentre, randomised controlled trial. Lancet.

[B9] Hentati H, Salloum C, Caillet P (2018). Risk factors for mortality and morbidity in elderly patients presenting with digestive surgical emergencies. World J Surg.

[B10] Inoue Y, Kawamoto A, Okugawa Y (2015). Efficacy and safety of laparoscopic surgery in elderly patients with colorectal cancer. Mol Clin Oncol.

[B11] Itatani Y, Kawada K, Sakai Y (2018). Treatment of elderly patients with colorectal cancer. Biomed Res Int.

[B12] Jeong SY, Park JW, Nam BH (2014a). Open versus laparoscopic surgery for mid-rectal or low-rectal cancer after neoadjuvant chemoradiotherapy (COREAN trial): survival outcomes of an open-label, non-inferiority, randomised controlled trial. Lancet Oncol.

[B13] Jeong SY, Park JW, Nam BH (2014b). Open versus laparoscopic surgery for mid-rectal or low-rectal cancer after neoadjuvant chemoradiotherapy (COREAN trial): survival outcomes of an open-label, non-inferiority, randomised controlled trial. Lancet Oncol.

[B14] Jeong SY, Park JW, Nam BH (2014c). Open versus laparoscopic surgery for mid-rectal or low-rectal cancer after neoadjuvant chemoradiotherapy (COREAN trial): survival outcomes of an open-label, non-inferiority, randomised controlled trial. Lancet Oncol.

[B15] Jiang SX, Wang XS, Geng CH (2009). Altering trend of clinical characteristics of colorectal cancer: a report of 3,607 cases. Ai Zheng.

[B16] Kang SB, Park JW, Jeong SY (2010a). Open versus laparoscopic surgery for mid or low rectal cancer after neoadjuvant chemoradiotherapy (COREAN trial): short-term outcomes of an open-label randomised controlled trial. Lancet Oncol.

[B17] Kang SB, Park JW, Jeong SY (2010b). Open versus laparoscopic surgery for mid or low rectal cancer after neoadjuvant chemoradiotherapy (COREAN trial): short-term outcomes of an open-label randomised controlled trial. Lancet Oncol.

[B18] Kitano S, Inomata M, Sato A (2005). Randomized controlled trial to evaluate laparoscopic surgery for colorectal cancer: Japan Clinical Oncology Group Study JCOG 0404. Jpn J Clin Oncol.

[B19] Lacy AM, Delgado S, Castells A (2008). The long-term results of a randomized clinical trial of laparoscopy-assisted versus open surgery for colon cancer. Ann Surg.

[B20] Lujan J, Valero G, Hernandez Q (2009). Randomized clinical trial comparing laparoscopic and open surgery in patients with rectal cancer. Br J Surg.

[B21] Miyasaka Y, Mochidome N, Kobayashi K (2014). Efficacy of laparoscopic resection in elderly patients with colorectal cancer. Surg Today.

[B22] Ouabdelmoumen A, Sbai A, Elmejjatti F (2018). Predictive factors of histological response after preoperative concomitant radiochemotherapy in middle and low rectal cancer. Asian Pac J Cancer Care.

[B23] Robinson CN, Balentine CJ, Marshall CL (2011). Minimally invasive surgery improves short-term outcomes in elderly colorectal cancer patients. J Surg Res.

[B24] Seshadri RA, Swaminathan R, Srinivasan A (2018). Laparoscopic versus open surgery for rectal cancer after neoadjuvant chemoradiation: Long-term outcomes of a propensity score matched study. J Surg Oncol.

[B25] She WH, Poon JT, Fan JK (2013). Outcome of laparoscopic colectomy for cancer in elderly patients. Surg Endosc.

[B26] Sobin LH, Compton CC (2010). TNM seventh edition: what’s new, what’s changed: communication from the International Union Against Cancer and the American Joint Committee on Cancer. Cancer.

[B27] Turrentine FE, Wang H, Simpson VB (2006). Surgical risk factors, morbidity, and mortality in elderly patients. J Am Coll Surg.

[B28] Van der Pas MH, Haglind E, Cuesta MA (2013a). Laparoscopic versus open surgery for rectal cancer (COLOR II): short-term outcomes of a randomised, phase 3 trial. Lancet Oncol.

[B29] Van der Pas MH, Haglind E, Cuesta MA (2013b). Laparoscopic versus open surgery for rectal cancer (COLOR II): short-term outcomes of a randomised, phase 3 trial. Lancet Oncol.

[B30] Yang Y, Wang HY, Chen YK (2020). Current status of surgical treatment of rectal cancer in China. Chin Med J (Engl).

